# miPEP31 alleviates Ang II-induced hypertension in mice by occupying Cebpα binding sites in the pri-miR-31 promoter

**DOI:** 10.1186/s12933-024-02337-5

**Published:** 2024-07-11

**Authors:** Xiangxiao Li, Hong Zhou, Pengfei Lu, Zilong Fang, Guangzheng Shi, Xinran Tong, Wendong Chen, Gonghao Jiang, Peili Zhang, Jingyan Tian, Qun Li

**Affiliations:** 1 The Department of Cardiovascular Medicine, State Key Laboratory of Medical Genomics, Shanghai Key Laboratory of Hypertension, Ruijin Hospital, Shanghai Institute of Hypertension,, Shanghai Jiao Tong University School of Medicine, 197 Ruijin 2nd Road, Shanghai, 200025 China; 2 Precision Research Center for Refractory Diseases, Institute for Clinical Research, Shanghai General Hospital, Shanghai Jiao Tong University School of Medicine, Shanghai, 200025 China; 3 Department of Endocrine and Metabolic Diseases, Shanghai Institute of Endocrine and Metabolic Diseases, Ruijin Hospital,, Shanghai Jiao Tong University School of Medicine, Shanghai, China; 4 Shanghai National Clinical Research Center for Metabolic Diseases, Key Laboratory for Endocrine and Metabolic Diseases of the National Health Commission of the PR China, Shanghai Key Laboratory for Endocrine Tumor, Clinical Trials Center, Ruijin Hospital,, Shanghai Jiao Tong University School of Medicine, Shanghai, China

**Keywords:** miPEP31, Angiotensin II, Blood pressure, T_reg_ cells, Cebpα

## Abstract

**Background:**

Previous studies have shown that peptides encoded by noncoding RNAs (ncRNAs) can be used as peptide drugs to alleviate diseases. We found that microRNA-31 (miR-31) is involved in the regulation of hypertension and that the peptide miPEP31, which is encoded by the primary transcript of miR-31 (pri-miR-31), can inhibit miR-31 expression. However, the role and mechanism of miPEP31 in hypertension have not been elucidated.

**Methods:**

miPEP31 expression was determined by western blot analysis. miPEP31-deficient mice (miPEP31^−/−^) were used, and synthetic miPEP31 was injected into Ang II-induced hypertensive mice. Blood pressure was monitored through the tail-cuff method. Histological staining was used to evaluate renal damage. Regulatory T (T_reg_) cells were assessed by flow cytometry. Differentially expressed genes were analysed through RNA sequencing. The transcription factors were predicted by JASPAR. Luciferase reporter and electrophoretic mobility shift assays (EMSAs) were used to determine the effect of pri-miR-31 on the promoter activity of miPEP31. Images were taken to track the entry of miPEP31 into the cell.

**Results:**

miPEP31 is endogenously expressed in target organs and cells related to hypertension. miPEP31 deficiency exacerbated but exogenous miPEP31 administration mitigated the Ang II-induced systolic blood pressure (SBP) elevation, renal impairment and T_reg_ cell decreases in the kidney. Moreover, miPEP31 deletion increased the expression of genes related to Ang II-induced renal fibrosis. miPEP31 inhibited the transcription of miR-31 and promoted T_reg_ differentiation by occupying the Cebpα binding site. The minimal functional domain of miPEP31 was identified and shown to regulate miR-31.

**Conclusion:**

miPEP31 was identified as a potential therapeutic peptide for treating hypertension by promoting T_reg_ cell differentiation in vivo. Mechanistically, we found that miPEP31 acted as a transcriptional repressor to specifically inhibit miR-31 transcription by competitively occupying the Cebpα binding site in the pri-miR-31 promoter. Our study highlights the significant therapeutic effect of miPEP31 on hypertension and provides novel insight into the role and mechanism of miPEPs.

**Supplementary Information:**

The online version contains supplementary material available at 10.1186/s12933-024-02337-5.

## Introduction

Peptide drugs play important roles in the treatment of diseases such as cancers [1], cardiovascular diseases [2], and immune diseases [3]. Approximately 100 peptide products have been approved for commercial use globally, and the number of these products will continue to increase in the future [4]. Compared to traditional antibodies and small molecule medicines, peptide therapy offers several advantages in terms of safety, effectiveness, and economic viability. Peptides can have good receptor subtype selectivity and less immunogenicity [3]. Therefore, identification of endogenously expressed peptides has become a new strategy for promoting the development of peptide drugs.

Hypertension is the most common chronic disease and is the leading risk factor for disability and premature death worldwide [5]. Both innate and adaptive immune responses play crucial roles in the development of hypertension and target organ damage [6]. The natriuretic peptide family plays a key role in blood pressure regulation [7]. However, there are almost no studies on endogenously expressed noncoding RNA-encoded peptides in the treatment of hypertension. In our recent research, we found that the peptide miPEP31 encoded by pri-miR-31 could inhibit miR-31 expression [8] and that miR-31 was involved in the negative regulation of hypertension and organ damage [9]. However, the role of miPEP31 in hypertension is unknown, and the mechanism of miPEP31 in hypertension has not been elucidated.

In this study, we aimed to verify the endogenous expression of miPEP31 in target organs and cells related to hypertension and explored the effect and mechanism of miPEP31 in Ang II-induced hypertensive mice.

## Methods

### Peptide sequences

miPEP31: MRDWASVSSLGSGLWKERLWKSITTKRDGIAPVTRNWRGGKMLA; scPEP: GWRTKDWSISPLKAVLGWIRTSGGDTRMNKLARLAKESWVRSMG. scPEP and miPEP31 have the same amino acid composition and quantity, with the only difference being that the sequence of amino acids in scPEP is randomly shuffled. The peptides were synthesized by ChinaPeptides. The molecular weight was confirmed by mass spectrometry, and the purity was greater than 95%.

### Antibody production

GL Biochem made a custom polyclonal antibody derived against miPEP31. Rabbits were immunized with a synthetic miPEP31 conjugated with KLH. Keyhole limpet hemocyanin (KLH)-conjugated antigen-adjuvant conjugates are used to enhance the production of antigen-specific antibodies in vaccine models by conjugating the antigen with a protein adjuvant. Sera were collected and affinity purified against the peptide immunogen.

### Animals

C57BL/6J (WT) mice (8–12 weeks of age) were purchased from Shanghai SLAC Laboratory Animal Co. miPEP31^−/−^ mice were donated by Professor Honglin Wang. The mice were used for all of the experiments according to the National Institutes of Health Guide for the Care and Use of Laboratory Animals with the approval (SYXK-2003-0026) of the Scientific Investigation Board of Shanghai Jiao Tong University School of Medicine in Shanghai, China. For amelioration of any suffering during these experimental studies, the mice were euthanized by CO_2_ inhalation.

### miPEP31^−/−^ mouse identification

Point mutation-bearing mice were generated using the CRISPR/Cas9 system. miPEP31^/−^ mice were identified by sequencing the PCR products from tail biopsies, and mice with point mutations in the ATG site (to ATT) of the miPEP31 coding region were selected for further study. A gRNA was cloned to target the predicted coding sequence of miPEP31. Cleavage efficiency was tested in cell culture, and then the gRNA and Cas9 mRNA were transcribed in vitro and spin-column purified. A plasmid containing miPEP31 coding region with point mutation at the “ATG” site was used to introduce homologous recombination. Mouse embryos were injected with an equal ratio (w/w) of gRNA and Cas9 mRNA and plasmid with point mutation allele into the pronucleus and cytoplasm and then transferred to a surrogate dam for gestation. F0 founders are expected to be mosaic, allelic disruption was confirmed in the F0 generation pups using sequencing of the PCR product on tail biopsies, and positive founders were bred to WT C57BL/6 mice to isolate potential point mutation alleles. Mutants in the F1 generation were identified by sequencing of the PCR product. One mouse line with point mutation on “ATG” site of miPEP31 coding region was chosen for further study. Forward and reverse primers were used for PCR identification. The primers used were as follows: CTGCCAAACATGCTAACCACAT (forward primer) and CAGATAAGCTGGTTCAAGAAAGAA (reverse primer). The reverse primer was used for reverse sequencing.

### Animal treatment and blood pressure measurement

Male and female mice were both used in the experiments. Half female and half male. Mice were anaesthetized via inhalation of 2% isoflurane. Mini-pumps (1002, Alzet, Cupertino, California) were implanted subcutaneously into mice to deliver Ang II (750 ng/kg per minute for intravenously injected peptide experiments or 550 ng/kg per minute for miPEP31^−/−^ experiments) or 0.9% NaCl. For the peptide treatment groups, the mice were intravenously injected with 50 µg of miPEP31 or scPEP on Day 1 after infusion and every 2 days. Blood pressure was measured by the tail-cuff method using a BP-2000 Blood Pressure Analysis System on Day 2 after infusion and every 2 days thereafter.

### Histological analyses

Kidney tissue fixed with paraformaldehyde was embedded in paraffin, and 6 μm sections were stained with haematoxylin and eosin (HE) or Masson’s trichrome. The percentages of fibrotic areas and glomerular areas were quantified using Image-Pro Plus and Adobe Photoshop. For the fibrotic percentage, we used Image-pro Plus to quantify. We Use the pipette in the segmentation tool to select the fibrotic part and click on it, then convert it into count value. Finally, the percentage is calculated. For the glomerular areas, we used Adobe Photoshop to quantify. Use the magnetic lasso tool to circle the glomeruli and count the pixel values. The glomerular area calculation is translated by the parameters of the microscope camera.

### Western blot

Protein was extracted from tissues (kidney, aorta, heart, and spleen) and cells using radioimmunoprecipitation assay (RIPA) buffer containing protease inhibitors. Then, the cells were lysed on ice for 20 min and centrifuged at 12,000 × g for 20 min at 4 °C, after which the supernatant was transferred to a new tube. The protein concentrations were determined using a BCA Protein Assay Kit (Thermo, Cat23225). Tricine sample buffer (Bio-Rad, Cat1610739) was used for the samples, and protein standards (Bio-Rad, Cat1610377) and 4-20% gels (Tanon, Cat180-8005) were used for WB analysis. Anti-miPEP31 (1:1000 dilution), anti-GAPDH (Proteintech, Cat60004-1-Ig, 1:10000 dilution), HRP-labelled goat anti-mouse IgG (H + L) (Beyotime cat. A0216, 1:2000 dilution) and HRP-labelled goat anti-rabbit IgG (H+ L) (Beyotime cat. A0208, 1:2000 dilution) these antibodies were used. The signal was detected with Immobilon Western HRP Substrate (Millipore, CatWBKLS0500) and an Amersham Imager 600 (GE Healthcare). The images were cropped for presentation.

### Quantitative RT‒PCR

Total RNA was extracted from mouse tissue or cultured cells with TRIzol Reagent (TaKaRa). Reverse transcription was performed using HiScript III All-in-One RT SuperMix Perfect (Vazyme, R333). qRT‒PCR was performed on an ABI Qs7 instrument using SYBR Green Mix (TaKaRa, RR420A). The relative expression of all genes was determined by normalization to that of the housekeeping gene GAPDH. The sequences of primers used were as follows:

GAPDH forward: TGTGTCCGTCGTGGATCTGA, GAPDH reverse: CCTGCTTCACCACCTTCTTGA; PPAR forward: GTAAATCTGCGGGATGATGG, PPAR reverse: GGTGGAAGCAGGGTCAAAA; PEPCK forward: ACACACACACATGCTCACAC, PEPCK reverse: ATCACCGCATAGTCTCTGAA; G6Pc forward: TGGTAGCCCTGTCTTTCTTTG, G6Pc reverse: TTCCAGCATTCACACTTTCCT; CDK2 forward: CCTGCTTATCAATGCAGAGGG, CDK2 reverse: TGCGGGTCACCATTTCAGC.

Runx1 forward: CACCGTCTTTACAAATCCGCCAC, Runx1 reverse: CGCTCGGAAAAGGACAAACTCC; Vtn forward: TGCTGCCTTCACTCGCATCAAC, Vtn reverse: GTCTGGTATGCCACTGAAGCCT; Col6a2 forward: TGGTCAACAGGCTAGGTGCCAT, Col6a2 reverse: TAGACAGGGAGTTGACTCGCTC; Tnxb forward: ATGAGGACCAGGTCACCATCTC, Tnxb reverse: GGCATCAGTAGGCTCCTCTTTG; Itga8 forward: CCGATTTGCTGTTCCTCGCCTT, Itga8 reverse: GACCTGAGCAATGGCAGTGATG; Grem2 forward: CTCGCCTTACAAGGATGGTAGC, Grem2 reverse: AGGTACTTGCGCTCGGTGACTA; Dcn forward: ACTCTCCAGGAACTTCGTGTCC, Dcn reverse: AGTCCCTGGAAGGCTCCGTTTT; Timp2 forward: AGCCAAAGCAGTGAGCGAGAAG, Timp2 reverse: GCCGTGTAGATAAACTCGATGTC; Fgfr2 forward: GTCTCCGAGTATGAGTTGCCAG, Fgfr2 reverse: CCACTGCTTCAGCCATGACTAC; Fgf9 forward: ACAGTGGACTCTACCTCGGCAT, Fgf9 reverse: GGTTGGAAGAGTAGGTGTTGTAC; Smad2 forward: CCAACTGTAACCAGAGATACGGC, Smad2 reverse: AACCCTGGTTGACAGACTGAGC; Smad3 forward: GCTTTGAGGCTGTCTACCAGCT, Smad3 reverse: GTGAGGACCTTGACAAGCCACT; Smad4 forward: CAGCCATAGTGAAGGACTGTTGC, Smad4 reverse: CCTACTTCCAGTCCAGGTGGTA; Shh forward: GGATGAGGAAAACACGGGAGCA, Shh reverse: TCATCCCAGCCCTCGGTCACT; Dll1 forward: GCTGGAAGTAGATGAGTGTGCTC, Dll1 reverse: CACAGACCTTGCCATAGAAGCC; Smurf1 forward: GGAGGAAGGTTTGGACTATGGTG, Smurf1 reverse: CCGTGGAATACTGGAAGAGTCC; Smurf2 forward: CCAATGCCATCAACCGCCTCAA, Smurf2 reverse: GTGCCTATTCGGTCTCTGGACT; Smad1 forward: CTGAAGCCTCTGGAATGCTGTG, Smad1 reverse: CAGAAGGCTGTGCTGAGGATTG; Smad5 forward: CAGGAGTTTGCTCAGCTTCTGG, Smad5 reverse: ACGTCCTGTCGGTGGTACTCTG; Smad6 forward: ACCAACTCCCTCATCACTGCTC, Smad6 reverse: AGCCTGGTCGTACACCGCATAG; Smad7 forward: GTCCAGATGCTGTACCTTCCTC, Smad7 reverse: GCGAGTCTTCTCCTCCCAGTAT.

For measurement of miR-31 expression, reverse transcription was performed using a TaqMan miRNA reverse transcription kit, miR-31 RT primers and U6 snRNA (Thermo, Cat4366596), and qRT‒PCR was performed using TaqMan probes for miR-31 and U6 snRNA (Thermo, Cat4427975). miR-31 expression was determined by normalization to endogenous U6 expression.

### Cell isolation, culture and differentiation

Splenocytes were obtained from 7-week-old mice, and naïve CD4^+^ T cells were enriched with a Naïve CD4 + Isolation Kit (STEMCELL Technologies, Cat19765). T cells were cultured in RPMI-1640 medium supplemented with 10% heat-inactivated foetal bovine serum (FBS), 2 mM L-glutamine, 1% antibiotic-antimycotic, 10 mM HEPES buffer, 1 mM sodium pyruvate, MEM Mon-Essential Amino Acids and 55 µM 2-mercaptoethanol (all from Gibco). The naïve T cells were activated with plate-bound anti-CD3 (5 mg/ml; BD Biosciences) and soluble anti-CD28 (2 mg/ml; BD Biosciences). T_reg_ cell differentiation was achieved by adding TGF-β1 (R&D Systems). All T cells were cultured for 3 days and detected by flow cytometry.

Kidneys were minced and placed in RPMI-1640 medium containing 1 mg/ml collagenase I (Gibco, Cat17100-017) at 37 °C for 30 min with shaking. Then, the cells were filtered through 40 μm cell strainers (Corning) to obtain single-cell suspensions. The cell suspension was subjected to Percoll (GE, Cat17089102) gradient centrifugation before flow cytometry.

### Flow cytometry

The surface markers and transcription factors were assessed using flow cytometry with LSRFortessa (BD Bioscience) or CytoFLEX (Beckman Coulter) and analysed with FlowJo software. The cells were fixed and permeabilized using fixation/permeabilization concentrate, diluent (eBioscience, Cat00-5123) and permeabilization buffer (eBioscience, Cat00-8333) according to the manufacturer’s instructions. The following antibodies were used: anti-mouse CD45 Alexa Fluor 700 (eBioscience, Cat56-0451-82), anti-mouse CD4 APC (eBioscience, Cat17-0041-83), anti-mouse Foxp3 PE (eBioscience, Cat12-5773-82), and Brilliant Violet 421 anti-mouse CD25 (BioLegend, Cat102043).

### Imaging flow cytometry

After staining, the cells were washed, fixed and analysed at a magnification of 40× via ImageStream flow cytometry (Amnis) and IDEAS analysis software (Amnis). In each sample, 60,000 events were collected and imaged in extended depth of field (EDF) mode. Digital spectral compensation was performed on a pixel-by-pixel basis using single-stained controls. The degree of miPEP31 nuclear translocation was analysed using similarity features, as described in IDEAS V.6.2.

### CCK-8 assay and annexin V-FITC/PI staining

NIH 3T3 cells were treated with different concentrations (0.1, 0.5, 1, 5, 10, or 50 µM) of miPEP31 or scPEP for 24 h. Then, according to the instructions of the CCK-8 Kit (APE×BIO), CCK-8 reagent was added to each well. After 4 h of incubation, the absorbance of each group was determined by an enzyme-labelled instrument at 450 nm, and the cell viability was calculated.

NIH 3T3 cells were treated with different concentrations (1, 10, 50 µM) of miPEP31 or scPEP for 24 h. The cells were collected and washed with PBS. According to the instructions for Annexin V-FITC/PI (Vazyme), the cells were resuspended in 100 µl of binding buffer, and then, 5 µl of Annexin V-FITC and 5 µl of PI were added and incubated in the dark for 5 min at room temperature. After that, 400 µl of binding buffer was added. Samples were detected by LSRFortessa (BD Bioscience) for 1 h.

### RNA interference

Small interfering RNA (siRNA) targeting mouse Cebpα was synthesized by GenePharma. Sense: GCGCAAGAGCCGAGAUAAATT; antisense: UUUAUCUCGGCUCUUGCGCTT. The sequences of the mouse miR-31 mimics were as follows: sense, AGGCAAGAUGCUGGCAUAGCUG; antisense, GCUAUGCCAGCAUCUUGCCUUU. The siRNA molecules were transfected using Lipo2000 (Invitrogen).

### Luciferase assays

NIH 3T3 cells were cultured in DMEM supplemented with 10% foetal bovine serum and 1% antibiotic-antimycotic. NIH 3T3 cells were seeded into 96-well plates (1 × 10^4^ cells per well) one day before transfection. The next day, the cells were cotransfected with 100 ng of the pGL3-promoter luciferase reporter vector and 10 ng of Renilla TK. For the siRNA groups, the siRNA working concentration was 80 nM. After 24 h of transfection, the cells were harvested for luciferase activity assays. Luciferase activity was assessed according to the Dual-Luciferase Reporter Assay protocol (Promega, CatE1910). Renilla TK luciferase activity was used to normalize the transfection efficiency.

The promoter regions were synthesized for cloning into the pGL3 sequence as follows:

WT-AATTACTTTAATAGTGACATGTTTGACTGCCGATTCTTATTTTTTGATCAAGGTAGTGGATCAACCTTGAGAGTTTTCAGTTCTTCAAAATGGAGTTCATGAGTCAGAGCCATTTTCAGG.

MUT-AATTACTTTAATAGTGACATGTTTGACTGCCGATTCTTATTTTTTGATCAAGGTAGTGGAATCCATTTGAGAGTTTTCAGTTCTTCAAAATGGAGTTCATGAGTCAGAGCCATTTTCAGG.

### Nuclear extraction and EMSA

Nuclear extracts were obtained from NIH 3T3 cells using the ProteinExt^®^ Mammalian Nuclear and Cytoplasmic Protein Extraction Kit (TransGen Biotech, CatDE201) according to the manufacturer’s instructions. Si-Cebpα-treated cells were harvested after 48 h of transfection. The gel shift assays were performed with a LightShift Chemiluminescent EMSA Kit (Thermo, Cat20148). According to the manufacturer’s recommendations, nuclear extracts (6 µg) and miPEP31 (200 pmol) were used. Anti-miPEP31 and anti- Cebpα antibody (Proteintech, Cat29388-1-AP) were used for the supershift. The mixture was incubated for an additional 20 min. The gel was prerun and run with 0.5% Tris-Borate-EDTA and processed according to the manufacturer’s instructions.

### RNA-seq and transcriptome analysis

miPEP31- or scPEP-treated hypertensive mice were euthanized 14 days after Ang II (750 ng/kg/min) infusion, after which kidney samples were isolated. miPEP31^−/−^ or WT hypertensive mice were euthanized 14 days after Ang II (550 ng/kg/min) infusion, and kidney samples were isolated. Total RNA was extracted with RNAiso Plus (Takara Bio) and purified with magnetic oligo (dT) beads after denaturation. Purified mRNA samples were reverse-transcribed into fragmented DNA samples and adenylated at the 3′ ends. Adaptors were ligated to construct a library. DNA was quantified by Qubit (Invitrogen). After cBot cluster generation, DNA samples were then sequenced by an Illumina HiSeq X Ten SBS instrument from OmicStudio. Raw data were converted into FASTQ format, and the number of transcripts per million fragments mapped (fragments per kilobase) was calculated and log2-transformed with Cuffnorm. Differential gene transcripts were analysed with DESeq.

### Statistical analysis

All the data are expressed as the means ± SEMs, and the statistical analysis was performed using GraphPad Prism 8. Student’s t test, one-way analysis of variance (ANOVA), or two-way ANOVA was performed to determine statistical significance. *P* < 0.05 was considered to indicate statistical significance. *P < 0.05; **P < 0.01; ***P < 0.001; ****P < 0.0001; ns, not significant. Error bars depict the SEM.

## Results

### miPEP31 deficiency exacerbates Ang II-induced hypertension and renal damage in mice

**Fig. 1:**
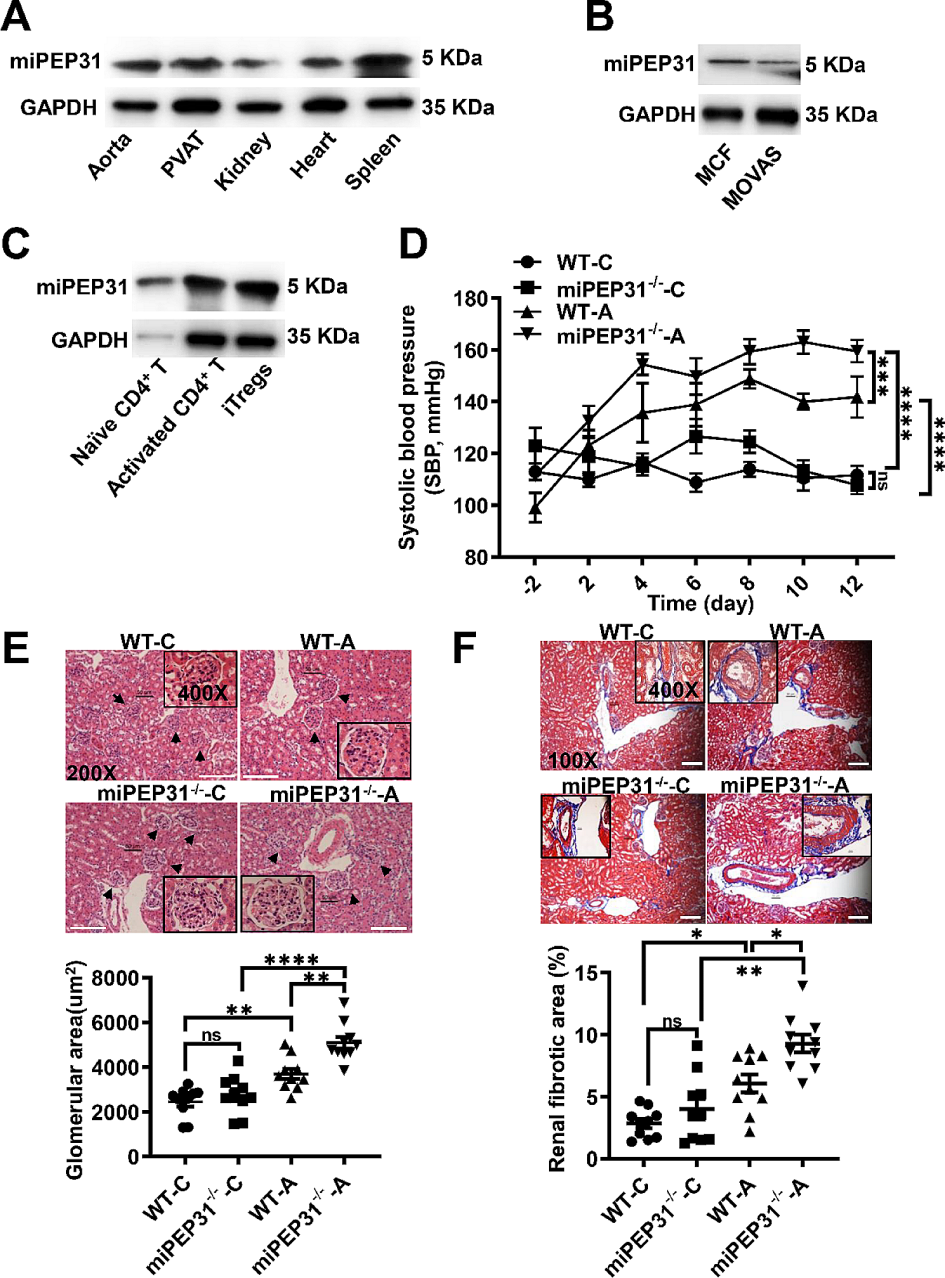
miPEP31 deficiency exacerbates Ang II-induced BP elevation and renal damage (**A**), Immunoblot analysis of the aorta, PVAT, kidney, heart, and spleen from WT mice with an anti-miPEP31 antibody (**B**), Immunoblot analysis of MCF and MOVAS cells (**C**), Immunoblot analysis of naïve CD4^+^ T cells, activated CD4^+^ T cells, and iT_reg_ cells from WT mice (**D**) , Construction of hypertensive mouse models in WT and miPEP31^−/−^ mice (550 ng/kg per minute Ang II infusion) for 2 weeks (WT-A, miPEP31^−/−^-A) and infusion with 0.9% NaCl as control group (WT-C, miPEP31^−/−^-C). Noninvasive tail cuff monitoring of systolic blood pressure (SBP) in the above 4 groups of mice (*n* = 7–10) (**E**). Representative haematoxylin and eosin (HE) staining of kidney sections and quantification of glomerular areas (*n* = 7–9). The black arrows indicate glomeruli; scale bar, 100 μm (**F**), Representative Masson’s trichrome staining of kidney sections, fibrotic tissues stained blue, and quantification of the percentage of renal fibrotic area (*n* = 10); scale bar, 100 μm. All the data are representative of three independent experiments. Statistical analyses were performed by two-way ANOVA (**D**, **E** and **F**). The data are expressed as the mean ± SEM. *P < 0.05, **P < 0.01, ***P < 0.001, ****P < 0.0001. *ns* indicates not significant

Similar to our previous studies [8], we showed that miPEP31 was endogenously expressed in hypertension-related tissues, such as the aorta, perivascular adipose tissue (PVAT), kidney, heart and spleen of wild-type (WT) mice (Fig. 1A), and cells, including mouse cardiac fibroblasts (MCF), mouse aortic vascular smooth muscle cells (MOVAS) (Fig. 1B), immune cells, naïve CD4^+^ T cells, activated CD4^+^ T cells, and iT_regs_ (induced regulatory T cells) (Fig. 1C), by using a polyclonal antibody against miPEP31 (Fig. S1A).

We generated a point mutant mouse strain (miPEP31^−/−^) in which the start codon in the ORF of miPEP31 was mutated from ATG to ATT by CRISPR/Cas9 (Fig. S1B). The sequencing results of the WT and miPEP31^−/−^ mice demonstrated that the mutation was successfully generated (Fig. S1C). And miPEP31 was depleted in miPEP31^−/−^ mice (Fig. S1D). Then, we constructed a hypertensive model in WT and miPEP31^−/−^ mice with 550 or 750 ng/kg per minute Ang II or 0.9% NaCl infusion and found that compared with WT mice, miPEP31^−/−^ mice exhibited a drastic increase in SBP and with averagely 18 mm Hg or 10 mm Hg higher SBP after 14 days of above 2 dose of Ang II in-fusion, respectively, SBP were increased in both miPEP31^−/−^ and WT mice in a dose-dependent manner during Ang II infusion, and there was no more pronounced difference significant comparing 550 to 750 ng/kg per minute after Ang II infusion, suggesting the optimum response was reached at 550 ng/kg per minute Ang II infusion (Fig. 1D, Fig. S1E). Renal damage is caused by Ang II–induced hypertension [10]. Therefore, we investigated the effect of miPEP31 deficiency on Ang II-induced kidney injury and found that miPEP31 deficiency accelerated Ang II-induced hypertrophy of glomeruli (Fig. 1E) by haematoxylin and eosin (HE) staining and increased renal fibrotic deposition by Masson’s trichrome staining (Fig. 1F). Thus, these data demonstrated that miPEP31 deficiency exacerbated Ang II-induced BP elevation and renal damage.

### **miPEP31 deficiency exacerbates the Ang II-induced T**_**reg**_**cell decrease and inflammation in the kidney**

**Fig. 2:**
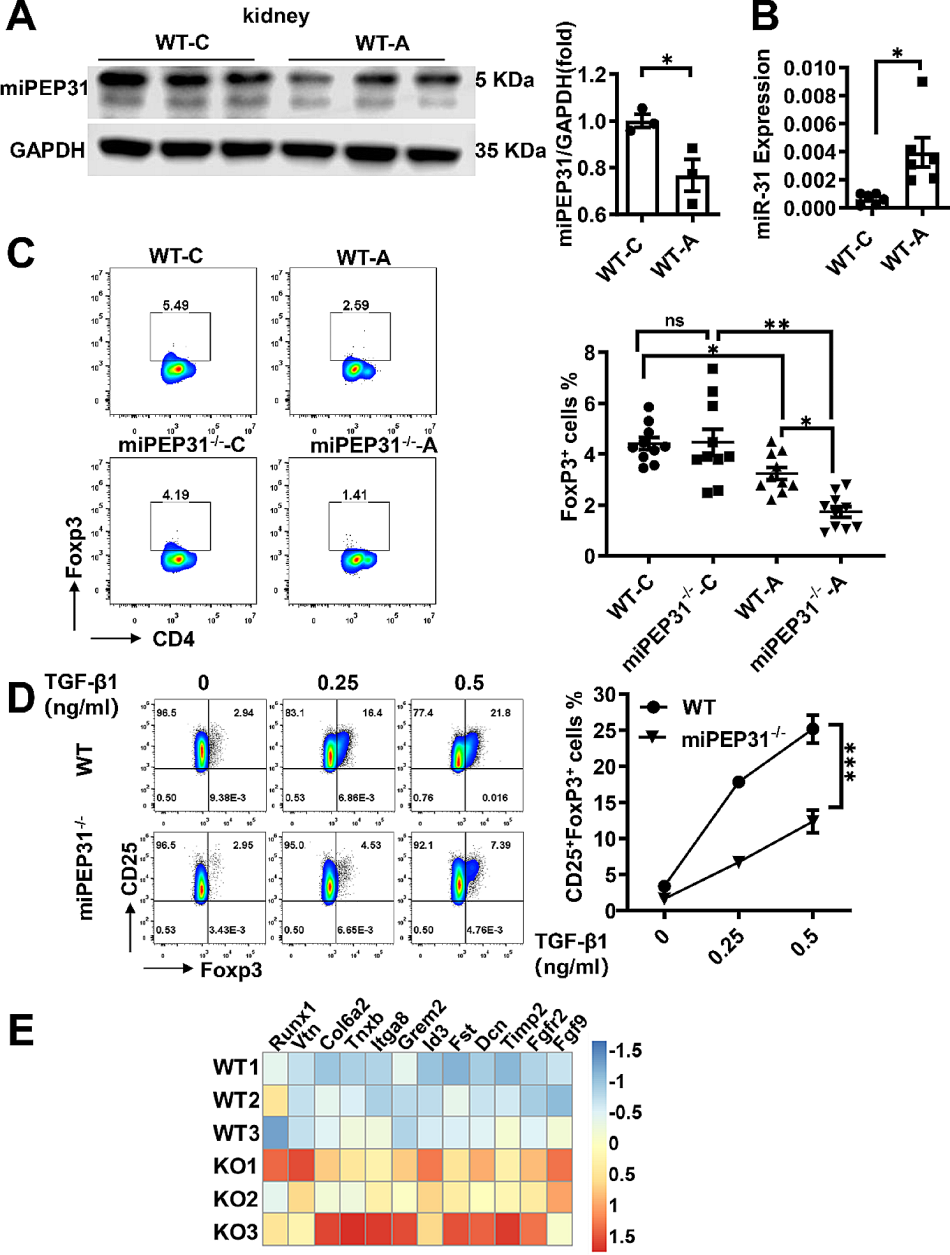
miPEP31 deficiency exacerbates Ang II-induced the T_reg_cells decrease and inflammation in the kidney (**A**) Immunoblot analysis of miPEP31 in the kidneys of C57BL/6 (WT-C) mice and Ang II-infused mice (WT-A). The quantification is presented as the ratio of miPEP31 to GAPDH (*n* = 3) (**B**), miRNA-31 expression in the kidneys of WT-A and WT-C mice was analysed by real-time quantitative PCR. The results are presented as the ratio of miRNA to the small nuclear RNA U6 (*n* = 6) (** C**), Representative flow cytometric analysis of CD4^+^Foxp3^+^ T_reg_ cells in kidney samples from the four groups and quantification of CD4^+^Foxp3^+^ T_reg_ cells (*n* = 6–8) (**D**), Naïve CD4^+^ T cells isolated from the spleens of WT and miPEP31^−/−^ mice. Flow cytometry of naïve CD4^+^ T cells induced to differentiate into T_reg_ cells in vitro with different concentrations of TGF-β1. The numbers in quadrants indicate the percentage of cells in each quadrant. Quantification of T_reg_ cell percentages (*n* = 6) (** E**), Heatmap of selected genes based on RNA-seq data from WT and miPEP31^−/−^ hypertensive mice on Day 15 after Ang II infusion. The colour key represents the normalized expression of genes. Statistical analyses were performed by two-tailed Student’s *t* test (**A** and **B**), two-way ANOVA (**C** and **D**). The data are expressed as the mean ± SEM. *P < 0.05, **P < 0.01, ***P < 0.001, ****P < 0.0001. *ns * indicates not significant

Here, we noted that miPEP31 expression was decreased but that miR-31 expression was significantly increased in the kidney from Ang II -infused for 14 days mice (WT-A) compared with those of WT control mice (WT-C) (Fig. 2A and B). To determine whether miPEP31 alters the number of T_reg_ cells in hypertensive mice, we showed that Ang II decreased the percentage of CD4^+^ FoxP3^+^ T_reg_ cells in the kidney, and this downregulation was notably exacerbated in the miPEP31^−/−^ mice compared with that in the WT mice (Fig. 2C). In vitro, we also found that the lack of miPEP31 markedly inhibited T_reg_ cell differentiation in a TGF-β1 dose-dependent manner in naïve CD4^+^ T cells from the miPEP31^−/−^ mice compared with those from the WT controls (Fig. 2D). These data suggested that miPEP31 deficiency negatively regulates in vitro–induced T_reg_ cell differentiation and exacerbates the Ang II–induced decrease in T_reg_ cells in the kidney. In addition, we performed RNA-sequencing analysis of kidney tissue from the Ang II-induced miPEP31^−/−^ (KO) mice and WT mice. The increased expression levels of renal fibrosis-related genes (*Grem2, Id3, Fst, Dcn*) and renal damage-related genes (*Runx1, Vtn, Clo6a2, Tnxb, Itga8, Timp2, Fgfr2, Fgf9*) further supported the idea that miPEP31 deficiency exacerbated Ang II-induced kidney inflammation (Fig. 2E). The results of qPCR were same with them (Fig.S2A).

### miPEP31 alleviates Ang II-induced BP elevation by promoting T_reg_ cell differentiation

**Fig. 3:**
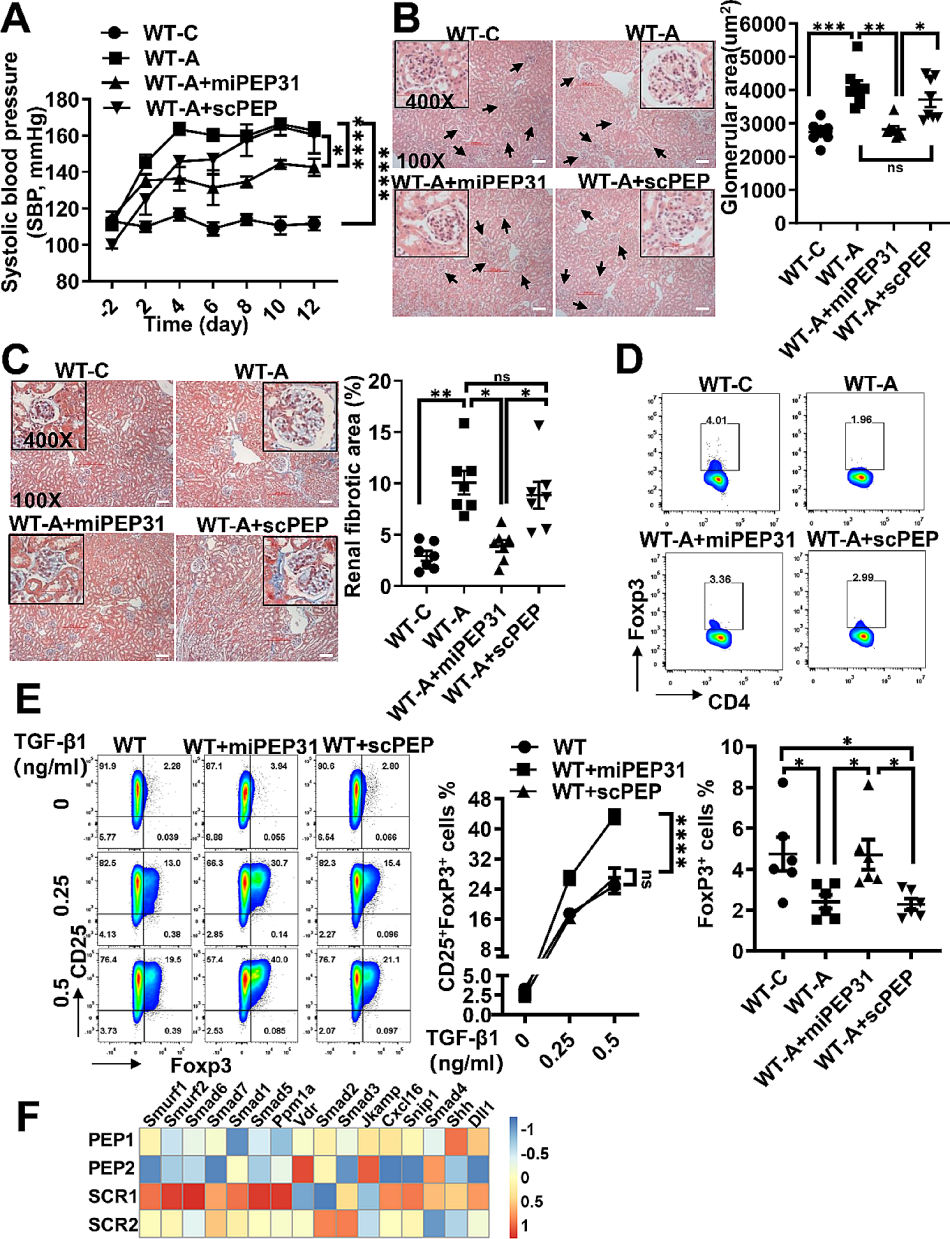
miPEP31 alleviates Ang II-induced BP elevation by promoting T_reg_cell differentiation (**A**), Construction of hypertensive mouse models in C57BL/6 (WT) mice (750 ng/kg per minute Ang II infusion for 2 weeks, WT-A group, and infusion with 0.9% NaCl, WT-C group). miPEP31 or scPEP (50 µg/per mouse) was injected intravenously every 2 days after Ang II infusion. Noninvasive tail cuff monitoring of systolic blood pressure (SBP) in the above 4 groups of mice (*n* = 6–9) (**B**), Representative haematoxylin and eosin (HE) staining of kidney sections and quantification of glomerular areas (*n* = 7–8). The black arrows indicate glomeruli; scale bar, 100 μm (**C**), Representative Masson’s trichrome staining of kidney sections, with fibrotic tissues stained blue, and quantification of the percentage of renal fibrotic area (*n* = 7–8); scale bar, 100 μm (**D**), Representative flow cytometric analysis of CD4^+^ Foxp3^+^ T_reg_ cells in kidney samples and quantification of CD4^+^ Foxp3^+^ T_reg_ cells (*n* = 6–8) (**E**) Naïve CD4^+^ T cells isolated from the spleens of WT mice. T_reg_ cells were induced in vitro with different concentrations of TGF-β1 and treated with 10 µM miPEP31 or scPEP. T_reg_ cells were detected by flow cytometry. Representative flow cytometric analysis of T_reg_ cells. Quantification of T_reg_ cell percentages (*n* = 6) (**F**), Heatmap of selected genes based on RNA-seq data from the kidneys of hypertensive mice treated with PEP (miPEP31) or SCR (scPEP) on Day 15 after Ang II infusion. The colour key represents the normalized expression of genes. Statistical analyses were performed by two-way ANOVA (**A**, **B**, **C**, **D** and **E**). The data are expressed as the mean ± SEM. *P < 0.05, **P < 0.01, ***P < 0.001, ****P < 0.0001. *ns* indicates not significant

Noninvasive tail cuff BP measurements revealed that miPEP31 treatment (WT-A+miPEP31) drastically blunted SBP elevation, while scPEP treatment (WT-A+scPEP) had no effect on SBP elevation compared with that in Ang II-induced hypertensive mice (WT-A) (Fig. 3A). A comparison of kidney sections from the WT-A +miPEP31, WT-A +sPEP and WT-A groups revealed that miPEP31 treatment restrained Ang II-induced glomerular hypertrophy and glomerular collagen deposition, but these effects were not observed in the scPEP treatment group (Fig. 3B and C). Flow cytometry analysis revealed that miPEP31 treatment ameliorated the Ang II-induced decrease in the percentage of CD4^+^ FoxP3^+^ T_reg_ cells in the kidney (Fig. 3D). Then, in vitro, we differentiated naïve CD4^+^ T cells into T_reg_ cells (WT) in the presence of synthetic miPEP31 (WT+ miPEP31) or control scPEP (WT+scPEP). We found that compared with the control, miPEP31 strongly promoted T_reg_ cell differentiation in a TGF-β1 dose-dependent manner, while scPEP had no effect on this induction (Fig. 3E). These results demonstrated that miPEP31 has therapeutic effects on hypertension by promoting the differentiation of T_reg_ cells.

We then investigated the gene expression profiles of kidneys derived from the miPEP31- or scPEP-treated hypertensive mice by performing RNA-sequencing analysis. The expression of genes that promote renal fibrosis (*Smad2, Smad3, Smad4, Shh, Dll1*) was downregulated, while the expression of genes that inhibit renal damage (*Smurf1, Smurf2, Smad1, Smad5, Smad6, Smad7*) was increased in the WT+miPEP31 group (PEP1/2) compared with the WT+ cPEP (SCR1/2) group (Fig. 3F). The change of above genes’ expression was consistent with the RNA-seq by qPCR (Fig. S2B). In addition, downregulated differentially expressed genes (DEGs) in the miPEP31-treated group were enriched in the complement activation pathway according to Metascape (Fig. S2C). Reducing complement activation was reported to alleviate hypertension [11]. These data suggested that miPEP31 mitigated Ang II-induced renal inflammation.

### miPEP31 promotes the differentiation of T_reg_ cells by competitively occupying the Cebpα binding site

**Fig. 4:**
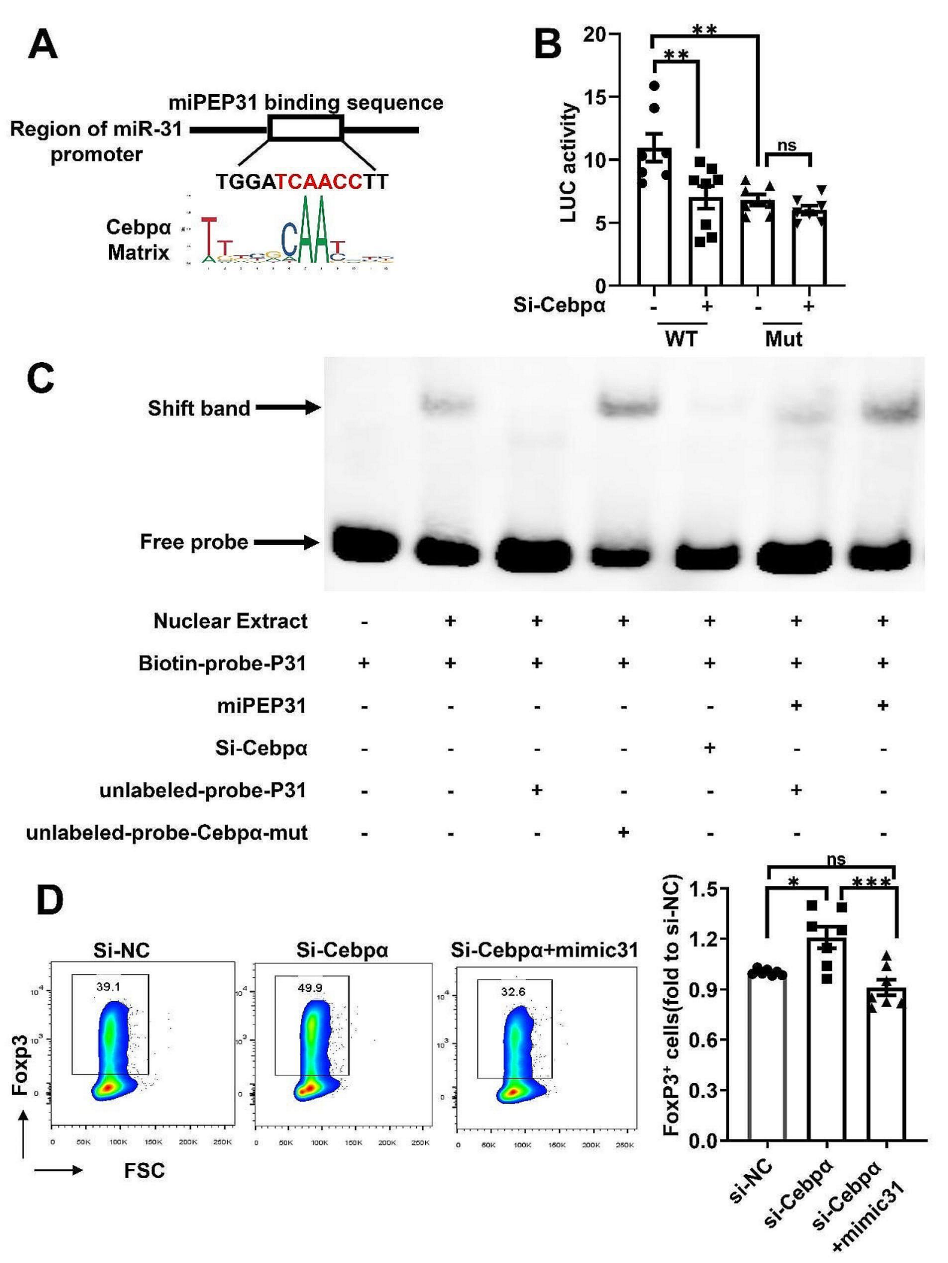
miPEP31 promotes the differentiation of T_regs_by competitively occupying the Cebpα binding site (**A**), The Cebpα binding site was predicted in the region of the pri-miR-31 promoter by JASPAR, and the Cebpα matrix is shown (**B**), Luciferase activity was measured in NIH 3T3 cells cotransfected with both PGL3-P31 (WT) or PGL3-P31 with the Cebpα binding site mutated (Mut) and Cebpα-siRNA (si-Cebpα) or negative control (si-NC) (*n* = 7–8) (**C**), EMSA to identify the interaction of P31 and miPEP31 or P31 with Cebpα. In some groups, nuclear extracts from NIH 3T3 cells transfected with Cebpα-siRNA were added. Nuclear extracts (6 µg) and miPEP31 (200 pmol) were used. The mixture was incubated for an additional 20 min. DNA‒protein complexes were separated upon migration on a native gel. Specific signals and free probes are indicated on the left side of the gel (**D**), Naïve CD4^+^ T cells derived from the spleens of WT mice were transfected with si-NC or si-Cebpα and mimic-31 to induce T_reg_ cells in vitro. Representative flow cytometric analysis of T_reg_ cells. Quantification of T_reg_ cell percentages (*n* = 7). Statistical analyses were performed by two-way ANOVA (**B**) or one-way ANOVA (**D**). The data are expressed as the mean ± SEM. *P < 0.05, **P < 0.01, ***P < 0.001, ****P < 0.0001. *ns * indicates not significant.

We used the JASPAR database to search for transcription factors (TFs) that could bind to the pri-miR-31 promoter and identified the predicted TFs [12] (Fig. S3A). We found a putative Cebpα binding site in the promoter region of pri-miR-31 (P31). The 6 bp core sequence of the Cebpα binding site is shown in Fig. 4A. Then, we generated reporter constructs that contained 6 bp core sequences of the Cebpα-binding site (WT) (TCAACC) or Cebpα-binding site-mutated (Mut) (ATCCAT) pri-miR-31 promoter. Silencing Cebpα in NIH3T3 cells harbouring the pri-miR-31 promoter (WT) decreased luciferase activity, and mutation of the 6 bp core sequence of the Cebpα binding site (Mut) decreased luciferase activity in parallel. As predicted, silencing Cebpα did not further reduce the luciferase activity of the Cebpα-binding site-mutated reporter construct (Fig. 4B), demonstrating that Cebpα transcriptionally regulates the expression of miR-31 through binding to its promoter region.

Accordingly, an EMSA showed that the DNA‒protein complexes (shift band) of the synthesized miR-31-promoter-derived probe (termed biotin-probe-P31) with nuclear extracts from NIH 3T3 cells were competitively inhibited by the addition of a cold probe (termed unlabelled probe-P31) or Cebpα siRNA, while after mutating the sequence of the Cebpα-related binding site (unlabelled probe-Cebpα-mut), a shift in the complex band was observed. We also verified that miPEP31 directly bound to the pri-miR-31 promoter (biotin-probe-P31) (Fig. 4C**)**. And the supershift bind demonstrated these bindings with miPEP31 or Cebpα (Fig. S3B and S3C). The data showed that miPEP31 could competitively bind to the Cebpα binding site of the pri-miR-31 promoter. Next, we investigated the function of Cebpα in the iT_reg_ differentiation system, which is negatively regulated by miR-31. The naïve CD4^+^ Tcells were transfected with Cebpα siRNA and differentiated into T_reg_ cells. The promotion of T_reg_ cell differentiation by Cebpα siRNA was reversed via transfection of the miR-31 mimic (Fig. 4D). In addition, we determined the expression of *PPAR* [13], *PEPCK, G6PC* [14], *CDK2*, and *CDK4* [15] (the downstream genes regulated by Cebpα) in NIH 3T3 cells stimulated with either miPEP31 or scPEP. The results showed that the miPEP31 and scPEP treatments did not have significantly different effects (Fig. S4A), even if the Cebpα binding site was identified in the promoter region of *PPAR, PEPCK, CDK2, CDK4* and *G6PC* (Fig. S4B). The results showed that the competitive occupation of the Cebpα binding site by miPEP31 affected miR-31 transcription but did not affect the expression of genes downstream of Cebpα (Fig. S4C).

### **miPEP31 is a cell-penetrating peptide that inhibits the increased expression of miR-31 in activated CD4**^**+**^**T cells**

**Fig. 5:**
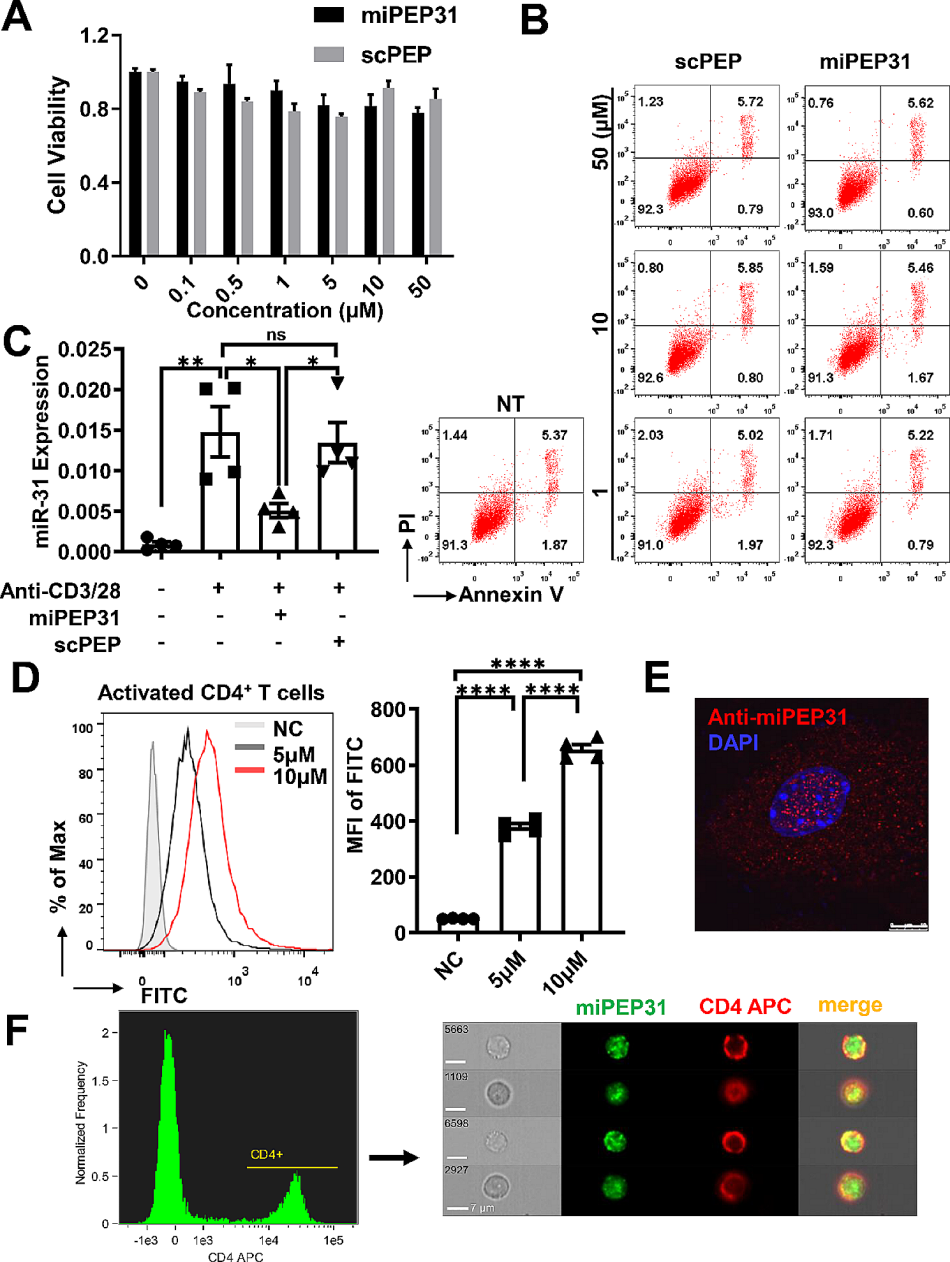
miPEP31 is a cell-penetrating peptide that inhibits the increased expression of miR-31 in activated CD4^**+**^T cells (**A**), The viability of NIH 3T3 cells cultured with different concentrations of miPEP31 or scPEP was evaluated via a CCK-8 assay. The values are expressed as fold changes relative to the controls (**B**), NIH 3T3 cells cultured with different concentrations of miPEP31 or scPEP. Flow cytometric analysis of the cell apoptosis rate in NIH 3T3 cells. The numbers in quadrants indicate the percentages of viable apoptotic and nonviable apoptotic cells (**C**), miRNA-31 expression in anti-CD3/28 -activated CD4^+^ T cells treated with 10 µM miPEP31 or scPEP was analysed by real-time quantitative PCR. The results are presented as the ratio of miRNA to the small nuclear RNA U6 (*n* = 4), (**D**) Activated CD4^+^ T cells (naïve CD4^+^ T cells with anti-CD3/28 antibodies) were treated with different concentrations of FAM-miPEP31 for 24 h, and the mean fluorescence intensity (MFI) was determined by flow cytometry. Quantification of FAM-miPEP31 expression (*n* = 4) (**E**), Representative immunofluorescence images of CD4^+^ T cells treated with 10 µM miPEP31 are shown; scale bar, 10 μm (**F**), C57BL/6 mice were injected intravenously with 100 µg of FAM-labelled miPEP31. Splenocytes were isolated and stained for CD4 and DAPI. CD4^+^ T cells were gated from the splenocytes of C57BL/6 mice. The expression of miPEP31 and CD4 was determined in CD4^+^ T cells by imaging flow cytometry; scale bar, 7 μm. Statistical analyses were performed by one-way ANOVA. The data are expressed as the mean ± SEM. *P < 0.05, **P < 0.01, ***P < 0.001, ****P < 0.0001. *ns * indicates not significant

It is important to determine the characteristics and functions of miPEP31 in CD4^+^ T cells. First, we investigated the effect of synthetic miPEP31 on cell viability and apoptosis. After NIH 3T3 cells were treated with the indicated doses of miPEP31 or its control (scPEP) for 24 h, cell viability and apoptosis were assessed by using CCK-8 and PI/Annexin V assays. The data showed that miPEP31 and scPEP had no obvious toxicity at concentrations up to 50 µM (Fig. 5A and B). Second, we found that the increased expression of miR-31 in activated CD4^+^ T cells stimulated with anti-CD3/28 was significantly inhibited by the addition of synthetic miPEP31 but not scPEP (Fig. 5C). Third, to clarify whether miPEP31 affects cells in a cell-penetrating manner, we incubated activated CD4^+^ T cells with fluorescein-labelled miPEP31 (FAM-miPEP31) and found that FAM-miPEP31 entered CD4^+^ T cells in a dose-dependent manner (Fig. 5D). The penetration of miPEP31 into CD4^+^ T cells was further confirmed by immunofluorescence imaging (Fig. 5E). In addition, 24 h after intravenous injection of 100 µg of FAM-labelled miPEP31 into WT mice, image flow cytometry detected miPEP31 in CD4^+^ T cells isolated from the spleen (Fig. 5F), indicating that miPEP31 could enter CD4^+^ T cells in vivo. Thus, miPEP31 could inhibit the increased expression of miR-31 in activated CD4^+^ T cells in a cell-penetrating manner without causing cell toxicity.

### The identified minimal functional domain of miPEP31 promotes T_reg_ cell differentiation

**Fig. 6:**
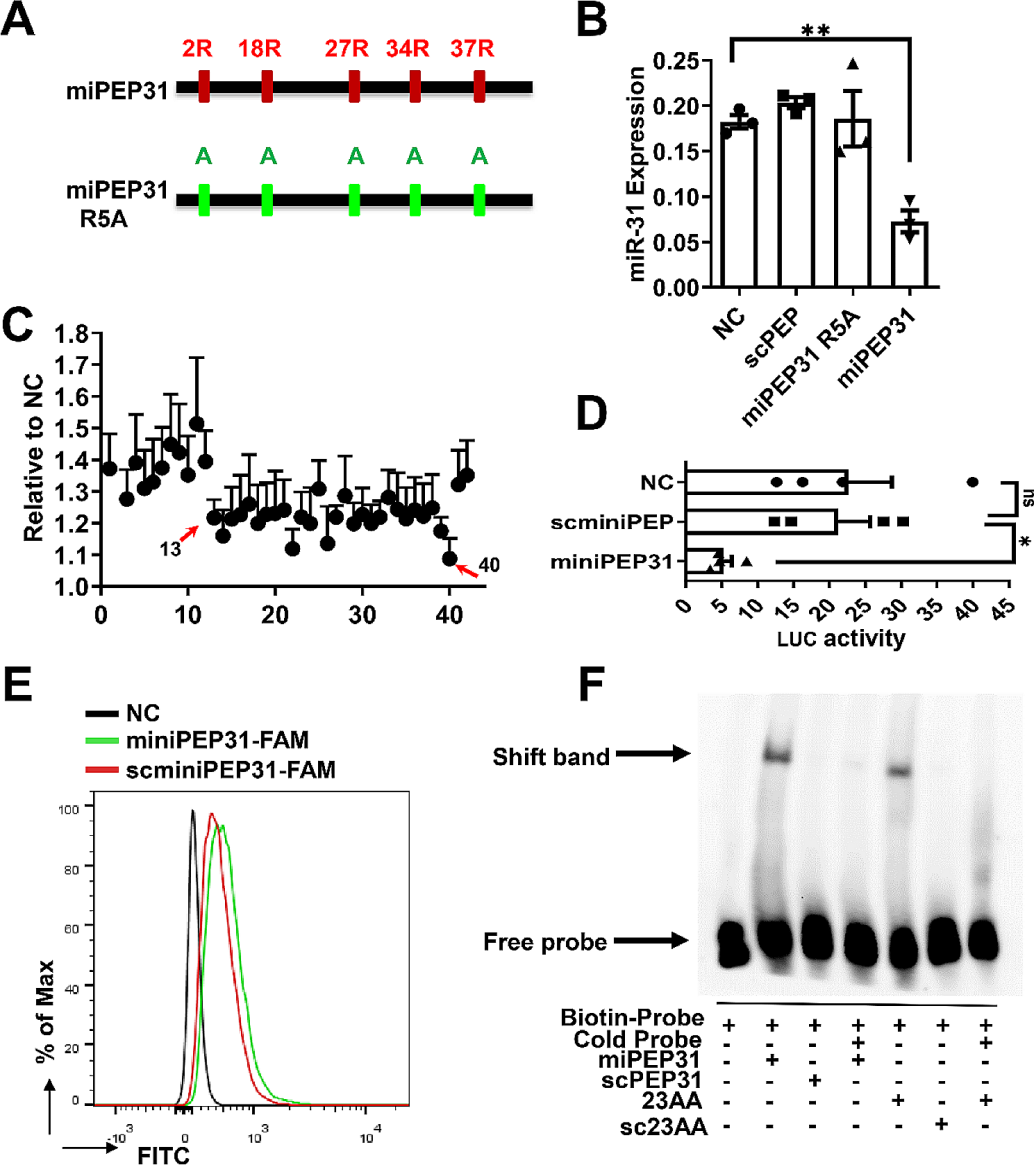
The identified minimal functional domain of miPEP31 promoted T_reg_cell differentiation. (**A**), Mutant Arginine to Alanine in miPEP31 to obtain the mutated miPEP31 (miPEP31 R5A).(**B**), miR-31 expression in NIH 3T3 cells treated with 10 µM scPEP, miPEP31 or miPEP31 R5A. The results are presented as the ratio of miRNA to the small nuclear RNA U6 (*n* = 3). (**C**), N-terminal or C-terminal cut peptides from miPEP31 were added to the polarizing medium for T_reg_ cell differentiation. Flow cytometry was used to analyse T_reg_ cell induction in the presence of different peptides. The values are expressed as fold changes relative to the nontreated control (*n* = 5–6). (**D**), Luciferase activity in NIH 3T3 cells transfected with luciferase reporter plasmids containing the pGL3 empty vector or the pri-miR-31 promoter and treated with miniPEP31 (from the 13th to the 42nd amino acid of miPEP31) or miniscPEP. The results are presented as the ratio of firefly luciferase activity to Renilla TK luciferase activity relative to that of untreated NIH 3T3 cells transfected with the pGL3 empty vector (*n* = 4). (**E**), Activated CD4^+^ T cells (naïve CD4^+^ T cells with anti-CD3/28 antibodies) treated with miniPEP31-FAM and scminiPEP31-FAM for 24 h were analysed by flow cytometry. (**F**), EMSA to identify the interaction between P31 and miPEP31 or miniPEP31. Nuclear extracts (6 µg) and miPEP31 or miniPEP31 (23AA) were used at 200 pmol. The cold probe competitively inhibited this process. scPEP31 and scminiPEP31 (sc23AA) were used as controls. The mixture was incubated for an additional 20 min. DNA‒protein complexes were separated upon migration on a native gel. Statistical analyses were performed by one-way ANOVA. The data are expressed as the mean ± SEM. *P < 0.05, **P < 0.01, ***P < 0.001, ****P < 0.0001. *ns * indicates not significant.

The minimum functional domain (MFD) of the peptide might be more effective and stable for potential therapeutic applications. Arginine is the critical amino acid required for the formation of DNA‒protein complexes because of the multiple hydrogen bonds formed between the amino acid side chain of arginine and DNA/RNA. Therefore, we mutated the key amino acid arginine (R) to alanine (A) in five domains, named miPEP31 R5A (Fig. 6A), and used it as a negative control to test its effect on miR-31 expression in NIH 3T3 cells treated with scPEP, miPEP31 R5A or miPEP31 (Fig. 6B). Next, we synthesized 42 peptides with amino acids 1–21 removed from either the N-terminus (referred to as amino acids 1 to 21) or the C-terminus (referred to as amino acids 42 to 22) of miPEP31. We added these 42 peptides to the polarizing medium of T_reg_ cells. Peptides lacking amino acids from the 13th to the 42nd residues exhibited a markedly reduced ability to induce T_reg_ cells, indicating that the peptide from the 13th to the 42nd amino acids of miPEP31 represents the MFD for T_reg_ cell induction (Fig. 6C). We named the peptide from the 13th to the 42nd amino acid miniPEP31. Luciferase reporter assays showed that miniPEP31 suppressed pri-miR-31 promoter activity (Fig. 6D). Furthermore, we used FAM-labelled miniPEP31 to treat activated CD4^+^ T cells and found that miniPEP31 was a cell-penetrating peptide (Fig. 6E). The EMSA results indicated that miniPEP31 bound to the nuclear protein to the same extent as miPEP31 (Fig. 6F). These results demonstrated that the identified miniPEP31 might play important roles through a molecular mechanism similar to that of miPEP31.

## Discussion

In this study, we found that miPEP31 deficiency exacerbated but that synthetic miPEP31 administration mitigated Ang II-induced SBP elevation and renal impairment by suppressing miR-31 expression and subsequently promoting iT_reg_ cell differentiation in mice. More importantly, miPEP31 promoted iT_reg_ cell differentiation by competitively occupying the Cebpα binding site in the pri-miR-31 promoter region. Our study revealed for the first time, to our knowledge, that the miRNA encoding the miPEP31 peptide is critical for treating hypertension and revealed the underlying mechanism involved.

Compared with traditional small molecules, peptide drugs, which have excellent safety, tolerability, and efficacy profiles, are potential therapeutic options. Moreover, the peptide production cost is much lower than that of small molecules [16]. The U.S. Food and Drug Administration has approved dozens of peptide-based drugs for the treatment of metabolic disorders and cancers [17]. miPEP-200a and miPEP-200b, which are encoded by pri-miRNAs (miR-200a and miR-200b), inhibit the migration of prostate cancer cells by regulating the epithelial-to-mesenchymal transition [18]. miPEP155 (encoded by the lnc miR155HG) alleviates psoriasis and EAE in two classic mouse models by modulating major histocompatibility complex class II-mediated antigen presentation and T-cell priming through disruption of the HSC70-HSP90 machinery [19]. ASRPS (encoded by LINC00908) acts as an antitumour peptide by reducing angiogenesis to significantly increase survival in patients with breast cancer [20]. Peptides can also act as molecular delivery vehicles for carrying drugs into cells.

Current treatments for hypertension include multiple therapeutic options, including angiotensin-converting enzyme inhibitors (lisinopril and captopril), angiotensin receptor blockers (losartan, valsartan, and olmesartan), calcium channel blockers (nifedipine, diltiazem, and verapamil), and diuretics (furosemide) [21]. However, the drug intolerance and side effects of these therapeutic options cannot be ignored [22]. Therefore, identification of effective drugs with excellent tolerability and safety is urgently needed. We identified miPEP31 as a potential therapeutic peptide for treating hypertension. miPEP31 acted as a transcriptional repressor to specifically inhibit miR-31 transcription by competitively occupying the Cebpα binding site in the pri-miR-31 promoter and promoting T_reg_ cell differentiation.

miPEP31 downregulates miR-31 expression, increases peripheral T_reg_ induction, and dramatically suppresses EAE [8]. Here, we demonstrated that miPEP31 expression is significantly decreased in kidney leukocytes from Ang II-induced hypertensive mice. Moreover, miPEP31 deficiency exacerbated and exogenous miPEP31 administration mitigated Ang II-induced increases in SBP and renal impairment and decreased the percentage of T_reg_ cells in the kidney. The data noted above indicate that the modulation of T_reg_ cell induction by miPEP31 may provide a basis for studies leading to potential therapies for hypertension. To investigate whether miPEP31 plays an important role in blood pressure (BP) regulation, we generated miPEP31^−/−^ mice and demonstrated that miPEP31 deficiency exacerbated Ang II-induced BP elevation and renal damage. The negative effect of miPEP31 deficiency on SBP led us to explore the potential therapeutic effect of miPEP31 in BP regulation. We verified this therapeutic effect by administering synthetic miPEP31 to an Ang II-induced hypertensive mouse model. Our results are the first to identify the regulatory role of miPEP31 in hypertension and suggest that this peptide could be used as a potential drug.

Our previous study revealed that miPEP31 can competitively bind to the enhancer region of pri-miR-31 and inhibit pri-miR-31 promoter activity at the transcriptional level and that miR-31 negatively regulates T_reg_ cell differentiation [8, 9]. Based on our above studies, miPEP31 has been confirmed to have a potential therapeutic effect on hypertension by promoting T_reg_ cell differentiation. To investigate how miPEP31 promotes T_reg_ cell differentiation by inhibiting miR-31 expression, we searched the JASPAR database to identify transcription factors (TFs) that could bind to the pri-miR-31 promoter. We found a putative Cebpα binding site in the promoter region of pri-miR-31 (P31), and Cebpα has been reported to affect the Th17/T_reg_ balance [23]. We demonstrated that Cebpα transcriptionally regulated the expression of miR-31 by binding to its promoter region. We further demonstrated that miPEP31 could competitively bind to the Cebpα binding site of the pri-miR-31 promoter. Taken together, these data suggest that miPEP31 specifically inhibits miR-31 expression by competitively occupying the Cebpα binding site within the pri-miR-31 promoter, thereby promoting the differentiation of T_reg_ cells.

Our study has several strengths. First, the role of miRNA-encoded peptides in hypertension has not been reported. For the first time, we explored the role of miPEP31 in a hypertensive mouse model, and point mutant mice were constructed for validation. Moreover, the mechanism of miPEP31 treatment in hypertensive mice was discovered. Although the effect and mechanism of miPEP31 in treating hypertension have been clarified, the findings of this study have several limitations. The BP detected by noninvasive tail cuff measurement was continuous and did not fully reflect the changes in BP. In fact, we detected BP at the same time of day. Whether other ncRNA-encoded peptides can also regulate hypertension should be explored. We look forward to further elucidating the clinical effect of miPEP31 in patients with hypertension.

In summary, our results confirmed that miPEP31 alleviates Ang II-induced hypertension in mice via T_reg_ cell accumulation, which highlights the significant therapeutic effect of miPEP31 on hypertension and provides novel insight into the effects and mechanism of miPEPs. These findings may provide future possibilities for the therapeutic prospects of peptides for cardiovascular diseases and diabetes.

### Supplementary material

Supplementary Material 1.

## Data Availability

The RNA-seq raw sequence data reported in this paper have been deposited in the Genome Sequence, Archivein National Genomics Data Center, China National Center for Bioinformation / Beijing Institute of Genomics, Chinese Academy of Sciences (GSA: CRA015213 and CRA015344) that are publicly accessible at https://ngdc.cncb.ac.cn/gsa.

## References

[1] Wang J, Zhu S, Meng N, He Y, Lu R, Yan GR. ncRNA-Encoded peptides or proteins and Cancer. Mol Therapy: J Am Soc Gene Therapy. 2019;27(10):1718–25. 10.1016/j.ymthe.2019.09.001 PMC6822234 31526596

[2] Kuwahara K. The natriuretic peptide system in heart failure: diagnostic and therapeutic implications. Pharmacol Ther. 2021;227:107863. 10.1016/j.pharmthera.2021.107863 33894277

[3] Muttenthaler M, King GF, Adams DJ, Alewood PF. Trends in peptide drug discovery. Nat Rev Drug Discovery. 2021;20(4):309–25. 10.1038/s41573-020-00135-8 33536635

[4] Rastogi S, Shukla S, Kalaivani M, Singh GN. Peptide-based therapeutics: quality specifications, regulatory considerations, and prospects. Drug Discovery Today. 2019;24(1):148–62. 10.1016/j.drudis.2018.10.002 30296551

[5] Lamirault G, Artifoni M, Daniel M, Barber-Chamoux N. Nantes University Hospital Working Group on H: resistant hypertension: Novel insights. Curr Hypertens Reviews. 2020;16(1):61–72. 10.2174/1573402115666191011111402 31622203

[6] Schiffrin EL. Inflammation, immunity and development of essential hypertension. J Hypertens. 2014;32(2):228–9. 10.1097/HJH.0000000000000042 24430118

[7] Rubattu S, Gallo G. The natriuretic peptides for Hypertension Treatment. High Blood Press Cardiovasc Prevention: Official J Italian Soc Hypertens. 2022;29(1):15–21. 10.1007/s40292-021-00483-5 34727352

[8] Zhou H, Lou F, Bai J, Sun Y, Cai W, Sun L, Xu Z, Liu Z, Zhang L, Yin Q et al. A peptide encoded by pri-miRNA-31 represses autoimmunity by promoting T(reg) differentiation. EMBO Rep 2022:e53475. 10.15252/embr.202153475 PMC9066071 35343645

[9] Li X, Cai W, Xi W, Sun W, Shen W, Wei T, Chen X, Sun L, Zhou H, Sun Y et al. MicroRNA-31 Regulates Immunosuppression in Ang II (Angiotensin II)-induced Hypertension by Targeting Ppp6C (Protein Phosphatase 6c). *Hypertension (Dallas, Tex*: 1979) 2019, 73(5):e14-e24. 10.1161/HYPERTENSIONAHA.118.12319 30929511

[10] Schmieder RE. End organ damage in hypertension. Deutsches Arzteblatt Int. 2010;107(49):866–73. 10.3238/arztebl.2010.0866 PMC3011179 21191547

[11] Chen XH, Ruan CC, Ge Q, Ma Y, Xu JZ, Zhang ZB, Lin JR, Chen DR, Zhu DL, Gao PJ. Deficiency of Complement C3a and C5a receptors prevents Angiotensin II-Induced Hypertension via Regulatory T Cells. Circul Res. 2018;122(7):970–83. 10.1161/CIRCRESAHA.117.312153 29437833

[12] Mathelier A, Fornes O, Arenillas DJ, Chen CY, Denay G, Lee J, Shi W, Shyr C, Tan G, Worsley-Hunt R, et al. JASPAR 2016: a major expansion and update of the open-access database of transcription factor binding profiles. Nucleic Acids Res. 2016;44(D1):D110–115. 10.1093/nar/gkv1176 PMC4702842 26531826

[13] Sekine K, Chen YR, Kojima N, Ogata K, Fukamizu A, Miyajima A. Foxo1 links insulin signaling to C/EBPalpha and regulates gluconeogenesis during liver development. EMBO J. 2007;26(15):3607–15. 10.1038/sj.emboj.7601784 PMC1949016 17627282

[14] Birney E, Stamatoyannopoulos JA, Dutta A, Guigo R, Gingeras TR, Margulies EH, Weng Z, Snyder M, Dermitzakis ET, Thurman RE, et al. Identification and analysis of functional elements in 1% of the human genome by the ENCODE pilot project. Nature. 2007;447(7146):799–816. 10.1038/nature05874 PMC2212820 17571346

[15] Wang GL, Iakova P, Wilde M, Awad S, Timchenko NA. Liver tumors escape negative control of proliferation via PI3K/Akt-mediated block of C/EBP alpha growth inhibitory activity. Genes Dev. 2004;18(8):912–25. 10.1101/gad.1183304 PMC395850 15107404

[16] Fosgerau K, Hoffmann T. Peptide therapeutics: current status and future directions. Drug Discovery Today. 2015;20(1):122–8. 10.1016/j.drudis.2014.10.003 25450771

[17] Zhang L, Eiden LE. Progress in regulatory peptide research. Ann N Y Acad Sci. 2019;1455(1):5–11. 10.1111/nyas.14260 PMC13119615 31646651

[18] Fang J, Morsalin S, Rao V, Reddy ES. Decoding of non-coding DNA and non-coding RNA: Pri-Micro RNA-Encoded novel peptides regulate Migration of Cancer cells. J Pharm Sci Pharmacol. 2017;3(1):23–7.

[19] Niu L, Lou F, Sun Y, Sun L, Cai X, Liu Z, Zhou H, Wang H, Wang Z, Bai J, et al. A micropeptide encoded by lncRNA MIR155HG suppresses autoimmune inflammation via modulating antigen presentation. Sci Adv. 2020;6(21):eaaz2059. 10.1126/sciadv.aaz2059 PMC7314557 32671205

[20] Wang Y, Wu S, Zhu X, Zhang L, Deng J, Li F, Guo B, Zhang S, Wu R, Zhang Z et al. LncRNA-encoded polypeptide ASRPS inhibits triple-negative breast cancer angiogenesis. J Exp Med 2020, 217(3). 10.1084/jem.20190950 PMC7062514 31816634

[21] Brouwers S, Sudano I, Kokubo Y, Sulaica EM. Arterial hypertension. Lancet (London England). 2021;398(10296):249–61. 10.1016/S0140-6736(21)00221-X 34019821

[22] Lobo MD, Sobotka PA, Pathak A. Interventional procedures and future drug therapy for hypertension. Eur Heart J. 2017;38(15):1101–11. 10.1093/eurheartj/ehw303 PMC5400047 27406184

[23] Zhang G, Wang W, Li S, Yang H, Zhang M, Zhang P, Wen Y, Wu A, Yang L, Zhou B, et al. IL6 gene allele-specific C/EBPα-binding activity affects the development of HBV infection through modulation of Th17/Treg balance. Genes Immun. 2015;16(8):528–35. 10.1038/gene.2015.40 26447433

[24] Chen T, Chen X, Zhang S, Zhu J, Tang B, Wang A, Dong L, Zhang Z, Yu C, Sun Y, et al. The genome sequence Archive Family: toward Explosive Data Growth and Diverse Data types. Genom Proteom Bioinform. 2021;19(4):578–83. 10.1016/j.gpb.2021.08.001 PMC9039563 34400360

[25] Database Resources of the National Genomics Data Center. China National Center for Bioinformation in 2022. Nucleic Acids Res. 2022;50(D1):D27–38. 10.1093/nar/gkab951 PMC8728233 34718731

