# Supplementary material for: miPEP31 alleviates Ang II-induced hypertension in mice by occupying Cebpα binding sites in the pri-miR-31 promoter
Source: Cardiovasc Diabetol. 2024 Jul 11;23:249. doi: 10.1186/s12933-024-02337-5 (PMC11241881; doi:10.1186/s12933-024-02337-5)
Supplement: Supplementary file 1 — Supplementary Material 1. [file 12933_2024_2337_MOESM1_ESM.docx]

**miPEP31 Alleviates Ang II-Induced Hypertension in Mice by Occupying Cebpα Binding Sites in the pri-miR-31 Promoter**

**Running title: miPEP31 Alleviates Hypertension**

Xiangxiao Li^1,2*^, Hong Zhou^2*^, Pengfei Lu^2^, Zilong Fang^1^, Guangzheng Shi^1^, Xinran Tong^1^, Wendong Chen^1^, Gonghao Jiang^1^, Peili Zhang^1^, Jingyan Tian^3,4#^, Qun Li^1#^

^1^ The Department of Cardiovascular Medicine, State Key Laboratory of Medical Genomics, Shanghai Key Laboratory of Hypertension, Ruijin Hospital, Shanghai Institute of Hypertension, Shanghai Jiao Tong University School of Medicine, Shanghai, 200025, China

^2^ Precision Research Center for Refractory Diseases, Institute for Clinical Research, Shanghai General Hospital, Shanghai Jiao Tong University School of Medicine, Shanghai 200025, China

^3^ Department of Endocrine and Metabolic Diseases, Shanghai Institute of Endocrine and Metabolic Diseases, Ruijin Hospital, Shanghai Jiao Tong University School of Medicine, Shanghai, China

^4^ Shanghai National Clinical Research Center for Metabolic Diseases, Key Laboratory for Endocrine and Metabolic Diseases of the National Health Commission of the PR China, Shanghai Key Laboratory for Endocrine Tumor, Clinical Trials Center, Ruijin Hospital, Shanghai Jiao Tong University School of Medicine, Shanghai, China

^*^ These authors contributed equally to this work

^#^ Correspondence: Dr. Jingyan Tian and Dr. Qun Li

Jingyan Tian, PhD

E-mail: tianjypaper@163.com

Qun Li, PhD

Shanghai Institute of Hypertension, Ruijin Hospital

Shanghai Jiao Tong University School of Medicine (SJTU-SM)

197 Ruijin 2nd Road

200025 Shanghai, China

E-mail: liqun@sibs.ac.cn


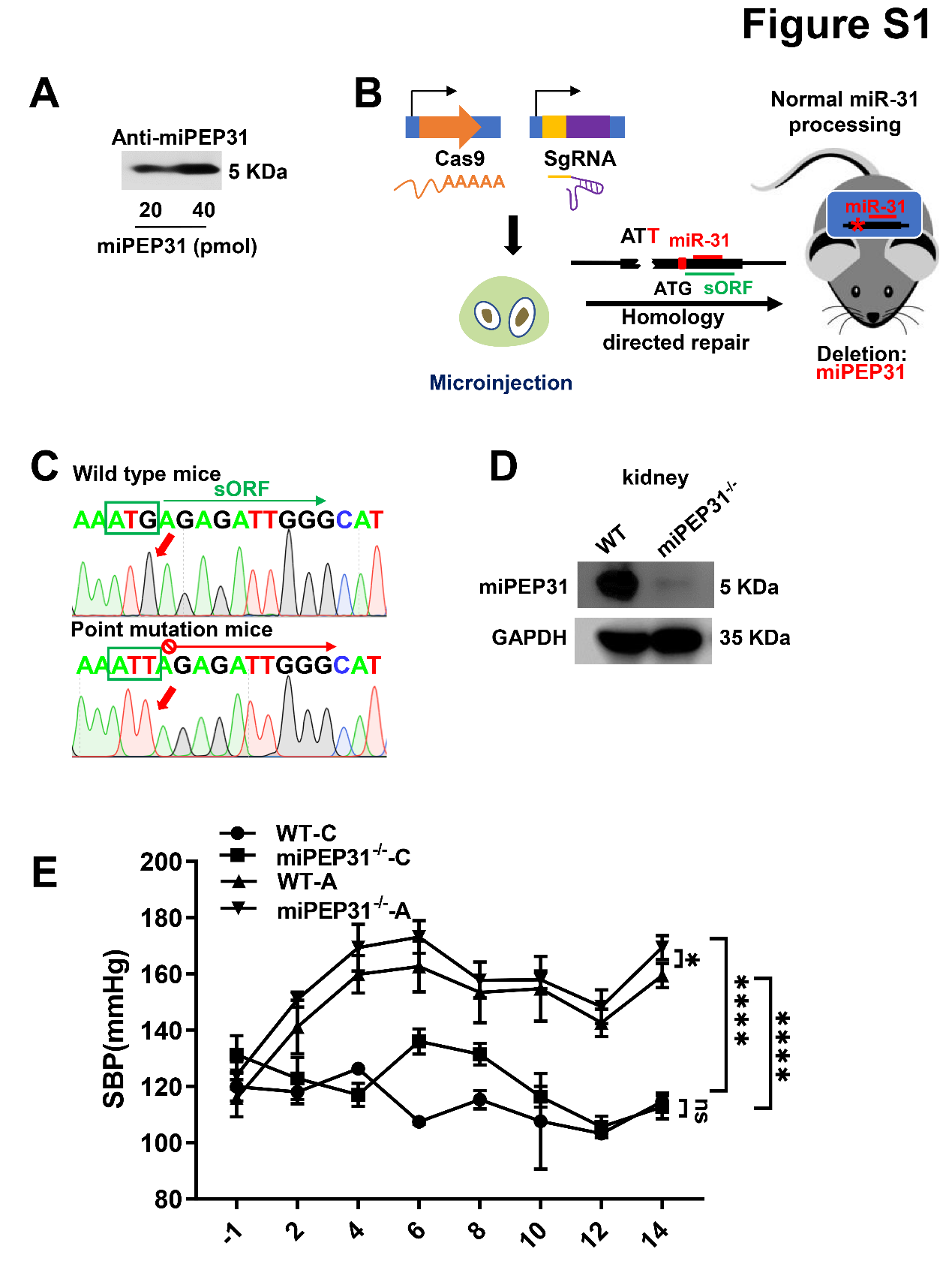
 **Figure S1. miPEP31^-/-^ mice generation and sequencing**

A, Immunoblot analysis of synthesized miPEP31 with specific miPEP31 antibody.

B, The point mutation mice (miPEP31^-/-^) were generation by CRISPR gRNA containing mutant sequence from ATG to ATT. All allele with a point mutation of ATT on the open read frame was chosen for further experiments.

C, The sequencing results of CRISPR/Cas9 targeted sequence in WT and the point mutation mice. The mutation is expected to be deficient to produce the miPEP31.

D, Immunoblot analysis of the kidney from WT mice and miPEP31^-/-^ mice with anti-miPEP31 antibody.

E, Construction of hypertensive mouse models in WT and miPEP31^-/-^ mice (750 ng/kg per minute Ang II infusion) for 2 weeks (WT-A, miPEP31^-/-^-A) and infusion with 0.9% NaCl as control group (WT-C, miPEP31^-/-^-C). Noninvasive tail cuff monitoring of systolic blood pressure (SBP) in the above 4 groups of mice (n=3-4). Statistical analyses were performed by two-way ANOVA (E). The data are expressed as the mean ± SEM. *P<0.05, **P<0.01, ***P<0.001, ****P<0.0001. ns indicates not significant.


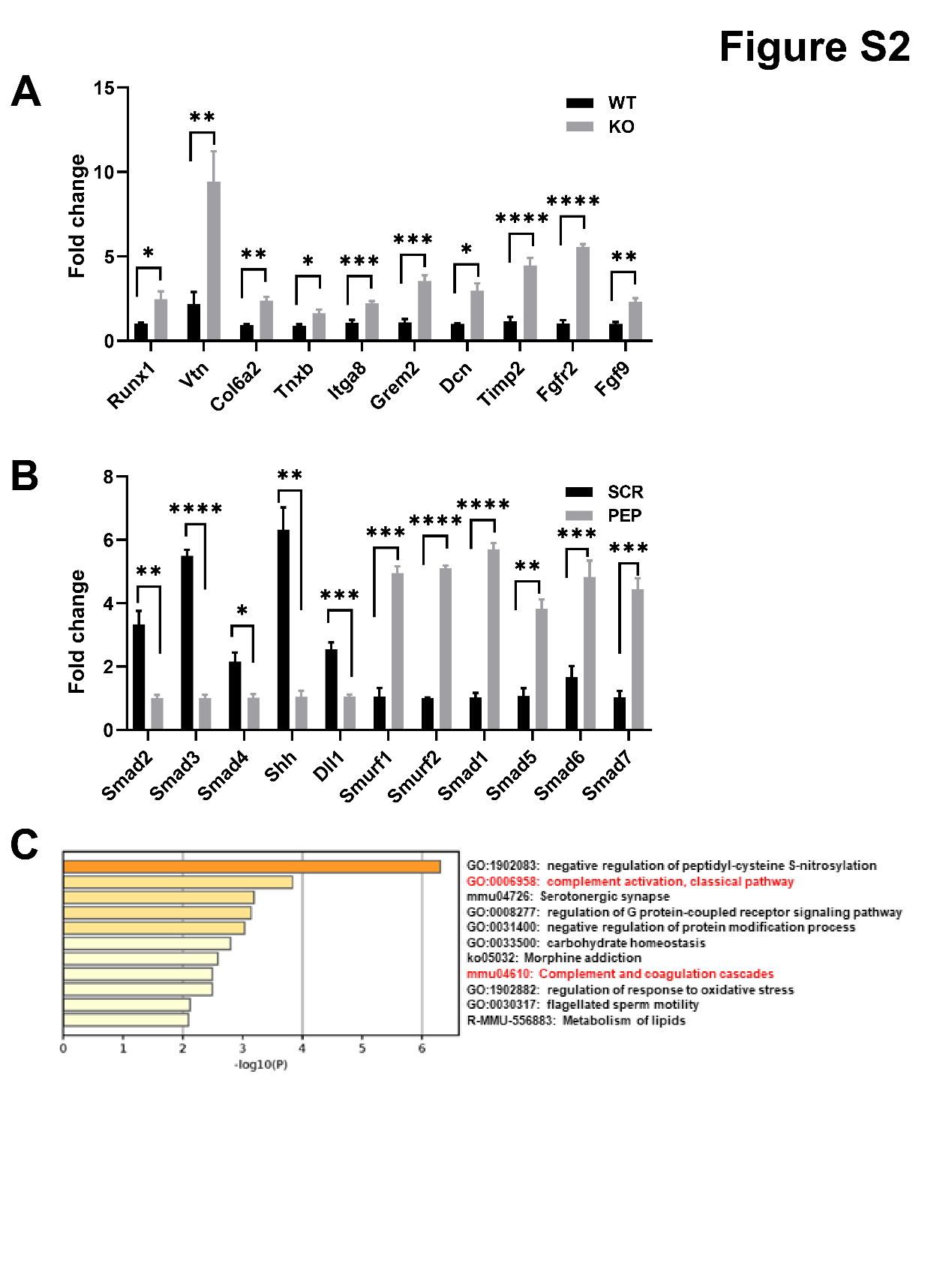
**Figure S2. Pathway enrichment analysis by Metascape**

A, Total RNAs of kidney samples derived from WT mice and miPEP31^-/-^ (KO) mice, the expression of Runx1,Vtn, Col6a2, Tnxb, Itga8, Grem2, Dcn, Timp2, Fgfr2 and Fgf9 was detected by qPCR. Results are presented as the ratio of GAPDH (n=3-6).

B, Total RNAs of kidney samples derived from miPEP31 (PEP) and scPEP (SCR) treated mice after Ang II infusion, the expression of Smad2, Smad3, Smad4, Shh, Dll1, Smurf1, Smurf2, Smad1, Smad5, Smad6 and Smad7 was detected by qPCR. Results are presented as the ratio of GAPDH (n=3-6).

C, Total RNAs of kidney samples derived from miPEP31 and scPEP treated mice after Ang II infusion were used for RNA-seq analysis. Pathway enrichment analysis of down-regulated DEGs in PEP (miPEP31) or SCR (scPEP) treated by Metascape. Statistical analyses were performed by two-tailed Student’s *t* test (A and C). The data are expressed as the mean ± SEM. *P<0.05, **P<0.01, ***P<0.001, ****P<0.0001. ns indicates not significant.


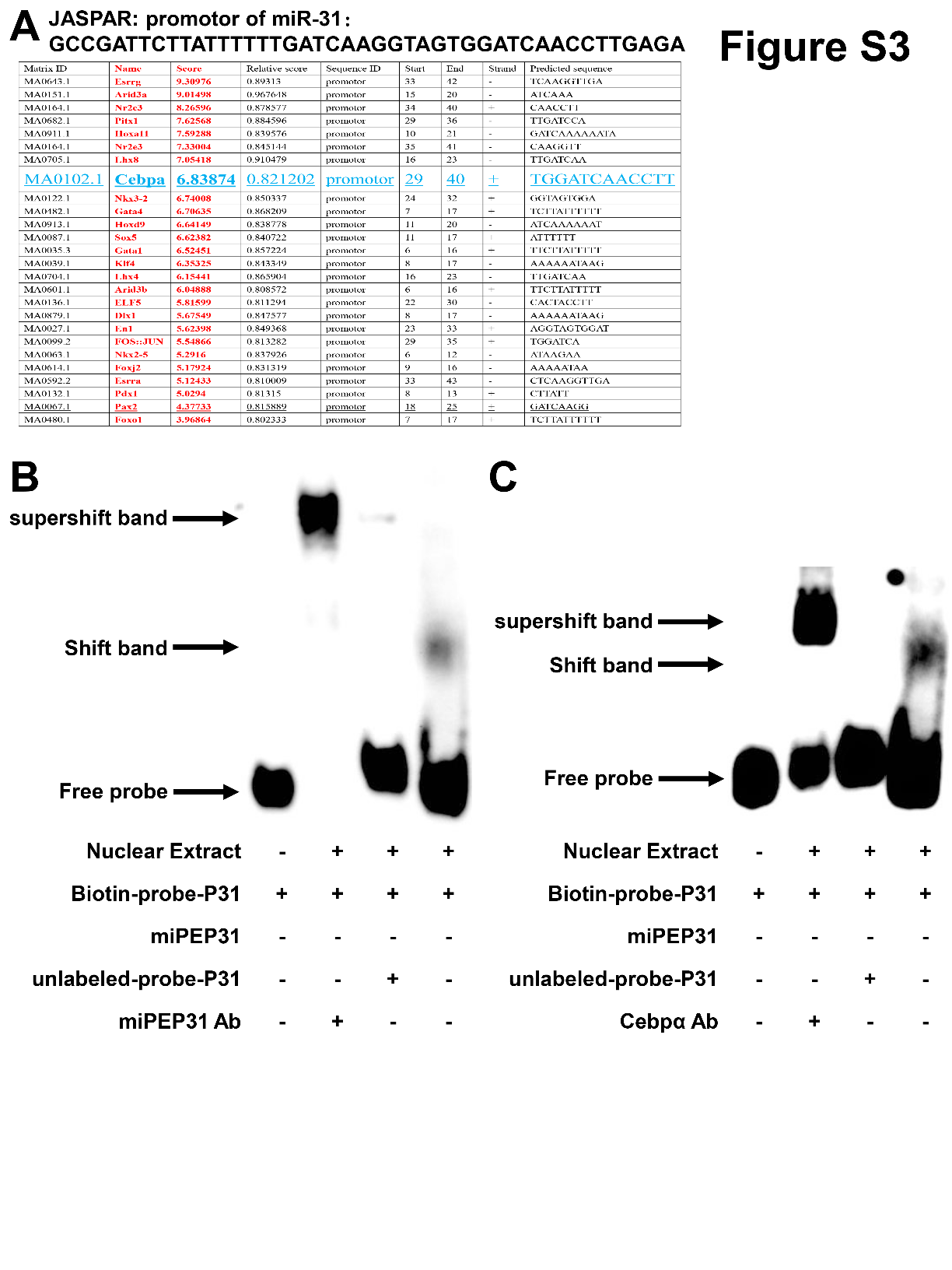
**Figure S3. Predict transcription factor binding to miR-31 promotor**

A, Find transcription factor binding to miR-31 promotor by JASPAR, the table showed the top TF.

B and C, EMSA to identify the interaction of P31 and miPEP31 or P31 with Cebpα. Nuclear extracts (6 μg), anti-miPEP31 antibody (2 μl) and anti-Cebpα antibody (2 μl) were used. The mixture was incubated for an additional 20 minutes. DNA‒protein complexes were separated upon migration on a native gel. Specific signals and free probes are indicated on the left side of the gel. Ab, antibody.


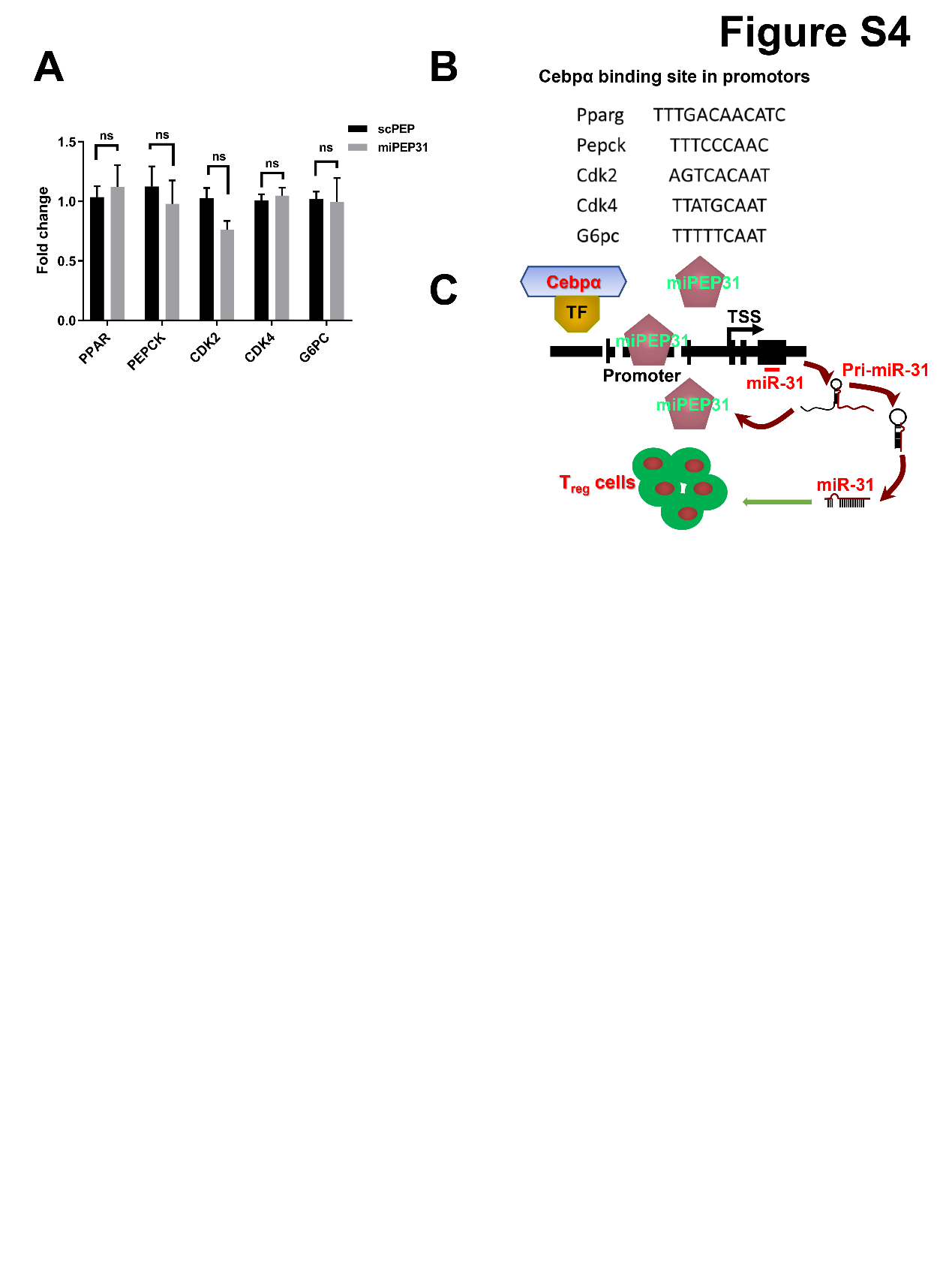
**Figure S4. The expression of Cebpα related genes**

A, NIH 3T3 cells were treated with 10 μM miPEP31 or scPEP, the expression of PPAR, PEPCK, CDK2, CD4, and G6PC was detected by qPCR. Results are presented as the ratio of GAPDH (n=4).

B, Cebpα binding site in the promoters of miR-31 and Cebpα related genes.

C, Schematic illustrating the mechanistic role of miPEP31. Statistical analyses were performed by two-tailed Student’s t-test. Data are expressed as mean ± SEM. *P<0.05, **P<0.01, ***P<0.001, ****P<0.0001. ns indicates not significant.
